# Malaria burden and treatment targets in Kachin Special Region II, Myanmar from 2008 to 2016: A retrospective analysis

**DOI:** 10.1371/journal.pone.0195032

**Published:** 2018-04-03

**Authors:** Hui Liu, Jian-Wei Xu, Yaw Bi

**Affiliations:** 1 Yunnan Institute of Parasitic Diseases, Yunnan Provincial Centre of Malaria Research, Yunnan Provincial Key Laboratory of Vector-borne Diseases Control and Research, Yunnan Provincial Collaborative Innovation Center for Public Health and Disease Prevention and Control, Pu’er City, China; 2 Laiza City Hospital, Laiza City, Kachin Special Region II, Myanmar; Agency for Science, Technology and Research - Singapore Immunology Network, SINGAPORE

## Abstract

Although drug-based treatment is the primary intervention for malaria control and elimination, optimal use of targeted treatments remains unclear. From 2008 to 2016, three targeted programs on treatment were undertaken in Kachin Special Region II (KR2), Myanmar. Program I (2008–2011) treated all confirmed, clinical and suspected cases; program II (2012–2013) treated confirmed and clinical cases; and program III (2014–2016) targeted confirmed cases only. This study aims to evaluate the impacts of the three programs on malaria burden individually based on the annual parasite incidence (API), slide positivity rate (SPR) and their relative values. The API is calculated from original collected data and the incidence rate ratio (IRR) for each year is calculated by using the first-year API as a reference in each program phase across the KR2. Same method is applied to calculate SPR and risk ratio (RR) at the sentinel hospital too. During program I (2008–2011), malaria burden was reduced by 61% (95%CI: 58%-74%) and the actual API decreased from 9.8 (95%CI: 9.6–10.1) per 100 person-years in 2008 to 3.8 (3.6–4.1) per 100 person-years in 2011. Amid program II (2012–2013), the malaria burden increased by 33% (95%CI: 22%-46%) and the actual API increased from 2.1(95%CI: 2.0–2.3) per 100 person-years in 2012 to 2.8 (95%CI: 2.7–2.9) per 100 person-years in 2013. During program III (2014–2016) the malaria burden increased furtherly by 60% (95%CI: 51% - 69%) and the actual API increased from 3.2(95%CI: 3.0–3.3) per 100 person-years in 2014 to 5.1 (95%CI: 4.9–5.2) per 100 person-years in 2016. Results of the slide positivity of the sentinel hospital also confirm these results. Resurgence of malaria was mainly due to *Plasmodium vivax* during program II and III. This study indicates that strategy adopted in program I (2008–2011) should be more appropriate for the KR2. Quality-assured treatment of all confirmed, clinical and suspected malaria cases may be helpful for the reduction of malaria burden.

## Introduction

Malaria is identified by the World Health Organization (WHO) in the World Malaria Report 2015 as a disease that continues to threaten human health globally [1]. In the year of 2015 alone, malaria caused 429 000 deaths and affected 212 million people [1]. The WHO plans to eliminate *P*. *falciparum* malaria by 2025 and turn all Greater Mekong Subregion (GMS) countries into non indigenous malaria by 2030 [2]. Drug-based treatment (henceforth calling treatment) is the primary interventional measure that is widely applied to fulfill successful malaria elimination [3]. However, optimal use of mass or targeted treatments remains unclear [3]. While the resistance of artemisinin and partner drug is also one of the factors that threatens the control and elimination of malaria, especially in the GMS [4], it is critical to accelerate malaria elimination before the failure of available antimalarial drugs.

Kachin Special Region II (KR2) is one of the malaria hot spots along China-Myanmar border (S1 Fig). Integrate-intervention strategy, including treatment, bednet distribution and health education, was adopted and implemented in KR2 from 2008 to 2016 but still end up on the losing side of malaria control [5]. Although a range of studies have been conducted in the KR2, few studies focused on malaria-treatment targets [6–11]. Three targeted programs on treatment were undertaken from 2008 to 2016. The sixth and tenth Rounds of China’s Global Fund to Fight AIDS, Tuberculosis and Malaria (GFATM) projects covered the KR2 from July 2007 to December 2013. From 2008 to 2011(Program I), the sixth round of China’s GFATM project aimed at treating all confirmed, clinical and suspected cases across the KR2. In 2012 and 2013(Program II), the first two years of the tenth round of China’s GFATM project turned to confirmed and clinical cases [5, 12]. The second phase of the tenth round of China’s GFATM project was consolidated into Global Fund New Funding Model (GFNFM) to intensify malaria control in Myanmar since 2014, and then from 2014 to 2016 (Program III), only malaria cases confirmed by microscopy or rapid diagnostic test (RDT) are eligible to be treated based on the guidelines for malaria treatment in Myanmar. To understand the impacts bringing by the three treatment programs, this paper offers the first publicly available retrospective analysis of the impacts of eight-year treatment since 2008.

## Methods

### Ethical statement

To explore the impacts of the three treatment strategies, this research uses a statistical analysis of routine data from malaria control programme. The Health Department of Kachin Special Region II of Myanmar has approved the access and collection of the data. All data has been fully anonymized before accessing. All data analysis processes have been approved by the Ethics Committees of the Yunnan Institute of Parasitic Diseases. In the case of anonymity, requirements on informed consent have been waived.

### Study site

The KR2 is one of the malaria hyperendemic areas in the northern Myanmar (S1 Fig). Total population of KR2 is about 60,000 and most people are Kachin Ethnic Minority (known as Jinghpaw in China). Hot climate, adequate precipitation and lush forest of KR2 provide a suitable environment for the growth and reproduction of mosquitoes and also for the transmission of malaria. With a complex vector community, *Anopheles dirus* and *Anopheles minimus* have been identified as two primary vectors [13]. In year-round malaria’s transmission, all of the parasite species (i.e. *P*. *falciparum*, *P*. *vivax*, *P*. *malariae* and *P*. *ovale*) exist in the area [7, 13]. To provide basic health service, Kachin Independent Organization (KIO) established a primary health care system with the support of international non-governmental organizations (NGOs). In the KR2, this primary health care system is continuously carrying out malaria control activities even in the context of war.

### Case definition and treatment

This study applies microscopy to detect parasites at malaria diagnosis and treatment stations (MDT). Rapid diagnostic test (RDT) is used by Village malaria workers (VMW), outreach teams and selected private clinics as a supplement. In this paper, a confirmed case is that of being tested as positivity by microscopic or RDT; a clinical case is the case that was not confirmed by any laboratory tests but with regular symptoms of fever, chills and sweating; and a suspected case is that the patient has a fever for less than 48 hours.

Confirmed *falciparum* malaria cases were treated with artemisinin-based combination therapy (ACT) for three days. ACT drugs used were mainly dihydroartemisinin- piperaquine (DP) (total dosage 2880mg [320mg D + 2560mg P] for an adult) from 2008 to 2013 and artemether-lumefantrine (total dosage 3360mg [480mg A+ 2880mg L] for an adult) from 2014 to 2016. Based on the Chinese guideline for malaria treatment, confirmed *vivax* malaria cases were given chloroquine (CQ) once a day for three days (total dosage 1200mg for an adult) and a total dosage 180mg of primaquine (PQ) once a day for eight days (22.5mg /day for an adult) from 2008 to 2013. The same dosage of CQ and a total dosage 210 mg of PQ for 14 days (15mg /day for a 50kg adult) were given during 2014–2016 according to the guideline of Myanmar. Infected children were dosed based on their body weight. From 2008 to 2013, ACT was administered for clinical and suspected cases in *P*. *falciparum* and mixed parasite prevalent areas accompanying with the adoption of CQ/PQ in single *P*. *vivax* prevalent areas. Although directly observed treatment (DOT) was not used, health staffs were required to inform caregivers or family members of patients to urge treatments.

### Data resources and collection

In this study, data was collected in two approaches: (1) Through health information system (HIS), established and maintained by the GFATM projects, number of malaria cases and treatment regimens administered monthly across the KR2 were captured [5, 12]; and (2) from the records of microscopy at Laiza City Hospital, which is the Headquarter Hospital of the KIO and also a sentinel hospital for malaria surveillance in the KR2.

### Statistical analysis

Annual parasite incidence (API) and slide positivity rate (SPR) were chosen as the indicators of malaria burden in our study. First, we calculated the API per 100 person-years with 95% confidence interval (CI). Second, we took the first-year API as the reference to calculate API rate ratios (IRR) with 95%CI for the following years of each program phase. Third, we worked out slide positivity rate (SPR) with 95%CI and took the first-year SPR as the reference to calculate risk ratio (RR) with 95%CI for microscopy’s data in sentinel hospital. Fourth, we adopted monthly slide positivity rates of the sentinel hospital to diagrammatize the seasonal fluctuation of malaria: 2006 representing for the malaria risk prior to systemic interventions of the GFATM projects, 2009 for treatment program I (2008–2011), 2013 for II (2012–2013) and 2015 for III (2014–2016). Finally, we combined the results of the IRR, RR and seasonal fluctuation of slide positivity to evaluate the impacts of the three treatment programs.

## Results

Total 88 547 treatment courses (52 552 ACT and 35 995 CQ/PQ) were administered during program I (2008–2011). Only in 2010, total 37 646 courses (22 457 ACT and 15 189 CQ/PQ) were delivered. We selected the API of the first year of this phase (2008) as the baseline of the phase. The IRR of the last year (2011) of this phase was 0.39 (0.36–0.42) that equates to a reduction of the malaria burden by 61% (95%CI: 58% - 74%) (S1 Table). The actual API was reduced from 9.8 (95%CI: 9.6–10.1) per 100 person-years in 2008 to 3.8 (3.6–4.1) per 100 person-years in 2011(S1 Table, S2 Fig). As the SPR of the first year (2008) of this phase was took as the control, the RR of the last year (2011) of this phase was 0.23 (95%CI: 0.19–0.28) that equates to a reduction by 77% (72% - 81%). The actual SPR of the sentinel hospital was decreased from 21.8% (95%CI: 19.4%-24.3%) in 2008 to 5.0% (95%CI: 4.2% - 5.9%) in 2011 (S2 Table).

Total 3 586 treatment courses (1 458 ACT and 2 128CQ/PQ) were administered during program II (2012–2013). As the API of the first year (2012) of this phase was used as the reference, the IRR of the last year (2013) was 1.33 (95%CI: 1.22–1.46) that equates to an increase of the malaria burden by 33% (95%CI: 22% - 46%) (S1 Table). The actual API increased from 2.1(95%CI: 2.0–2.3) per 100 person-years in 2012 to 2.8(95%CI: 2.7–2.9) per 100 person-years in 2013 (S1 Table, S2 Fig). As the SPR of the first year (2012) was took as the control, the RR of the last year (2013) was1.27 (95%CI: 1.04–1.56) that equates to an increase of 27% (95%CI: 4% - 56%) (S2 Table). The actual SPR of the sentinel hospital increased from 5.7% (95%CI: 4.8% - 6.7%) in 2012 to 7.3% (95%CI: 6.4% - 8.2%) in 2013 (S2 Table).

Total 7 521 treatment courses (8 85 ACT and 6 636CQ/PQ) were administered during program III (2014–2016). As the API of the first year (2014) of this phase was used as the baseline, the IRR of the last year (2016) was 1.60 (95%CI: 1.51–1.69) that equates to an increase of the malaria burden by 60% (95%CI: 51% - 69%) (S1 Table). The actual API increased from 3.2(95%CI: 3.0–3.3) per 100 person-years in 2014 to 5.1 (95%CI: 4.9–5.2) per 100 person-years in 2016 (S1 Table, S2 Fig). As the SPR of the first year (2014) was used as the control, the RR associated with slide positivity of the last year (2016) was 3.02 (95%CI: 2.70–3.39) that equates to an increase of 202% (170% - 339%). The actual SPR of the sentinel hospital increased from 8.4% (95%CI: 7.5% - 9.3%) in 2014 to 25.3% (95%CI: 24.1% -26.5%) in 2016 (S2 Table).

The total incidence of *P*. *falciparum* malaria decreased from 3 827 (67.0% of total confirmed cases, 95%CI: 65.7%-68.2%) in 2008 to 134 (4.1%, 95%CI: 3.4%-4.8%) in 2016 (S1 Table). The slide positivity number of *P*. *falciparum* reduced from 64 (25.9% of total slide positivity, 95%CI: 20.6% -31.8%) in 2008 to 19 (1.5%, 95%CI: 0.9% - 2.4%) in 2016 at the sentinel hospital (S2 Table). Comparing with 2006 (prior to the systemic interventions), the slide positivity rates of the sentinel hospital decreased in most of the months of 2009 (program I), 2013(program II) and 2015(program III). The months for slide positivity peak were March (48.9%) and June (49.3%) in 2006 (two peaks), July (32.5%) in 2009, June (19.2%) in 2013 and May (30.9%) in 2015. Although the monthly distribution of peaks above shows that the peak is shrinking and moving from 2006 to 2015, the slide positivity rates were resurging again from 2012 to 2016 (S2 Table, S3 Fig).

## Discussion

The purpose of this paper is to evaluate the impacts of the three targeted treatment programs on malaria burden across the KR2, Myanmar. Malaria burden was reduced by 61% during treatment program I (2008–2011) that targeted all confirmed, clinical and suspected cases. The incidence of *P*. *falciparum* malaria continuously decreased but the overall malaria burden increased because of increasing *P*. *vivax* incidence during treatment program II (2012–2013) that targeted confirmed and clinical cases and program III (2014–2016) that only targeted confirmed cases. The failure of the program II and III indicates the challenges in treatment and control of *P*. *vivax*.

In western Thailand, the results of quantitative-PCR documented that more than 90% of *Plasmodium spp*. infections were asymptomatic and submicroscopic within the studied community, where a high proportion of clinically febrile patients were misdiagnosed and undertreated [14, 15]. Accurateness of microscopy largely relies on the capacity, carefulness and responsibilities of microscopists. The best expert microscopists have the ability of a detection limit > 4 p/ul and the capacity of most expert microscopists should be > 30 p/ul. RDT has limitations in specificity, sensitivity and quality. The detection limit of RDT is between 100 and 200 p/ul and current mainstream available RDT is more sensitive in detecting *P*.*falciparum* than detecting *P*. *vivax*. The PCR can detect < 4 p/ul but is difficult to operate in the field [16–19]. Thus, the application of RDT in the program III (2014–2016) suggests that a high proportion of *Plasmodium spp*. infections may be missed. Another problem is that most cases do not have regular symptoms of fever, chills and sweating [20]. Therefore, the same story may happen in the program II (2012–2013). Passive detections and treatments are not sufficient for the quickly elimination of malaria because they cannot detect asymptomatic carriers. However, submicroscopic and asymptomatic carriers could still be treated if they have clinical attacks. Thus, immediate administration of antimalarial drugs to all confirmed, clinical and suspected cases can help cut off the transmission of malaria. As with the help of other interventions, malaria transmission can be interrupted finally. To eradicate malaria, administration of antimalarial drugs to all confirmed, clinical and suspected cases should be considered into the treatment strategies in hyperendenic areas like the KR2. On the other hand, comparing with mass drug administration (MDA), this strategy may also help reduce the risk of drug’s adverse effects and the cost of drugs together with their administration.

High coverage of malaria intervention is crucial at any stage of malaria control and elimination [21]. Malaria elimination is steadily implemented in China now [22]. In 2016, only three indigenous malaria cases were reported in the region of China by adopting high coverage of treatment programs. In Jiangsu Province, from 1973 to 1983, MDA with pyrimethamine and primaquine was used extensively and with up to 30 million people in target counties covered in a peak year (50% of the population size). Thus, the annual incidence has decreased 56.7% (95% CI: -75.5% to -23.7%) from 1973 to1976 and decreased 12.4% (95% CI: -24.7% to 2.0%) from 1976 to 1983 [23]. Yunnan Province used to be an important malaria endemic area. From 2006 to 2009, treatment targeted all confirmed, clinical and suspected malaria cases. A total of 334 316 courses (56 272 ACT and 278 044 CQ/PQ) were administered to febrile patients. The number of the courses is 13.3 times of the confirmed malaria cases (in total 25 070). Besides, a total of 250 456 PQ eight-day courses were provided for radical cure treatment in low transmitting season. As a result, the API was reduced by 77.9%, from 2.80 per 10 000 person-years in 2006 to 0.62 per 10 000 person-years in 2009[24]. Since 2010, China has switched from malaria control to elimination. In 2010, assuming there is no repeat medication within individuals, we administered 37 646 courses (22 457 ACT and 15 189 CQ/PQ) in the KR2, which is 62.6% (95%CI: 62.2–63.0) of the total population (i.e. 60 123). The API was reduced by 50%, from 6.4 (95%CI: 6.2–6.6) per 100 person-years in 2009 to 3.2 (3.1–3.4) per 100 person-years in 2010 (S1 Table, S2 Fig). This success also documented the importance of high access and coverage of treatment in malaria control.

The population size, genetic diversity, multi-clonal infections and transmission intensity of malaria parasites are associated with the spread of anti-malarial resistance [25]. Resistance is most likely to occur in patients with high numbers of parasites [26]. Not only can quality-assured diagnosis and treatment help patients improving outcome, but also limit the drug resistance [27]. The ACT watch group reported that the structure of anti-malarial market, constituted by community health workers, general retailers, itinerant drug vendors, pharmacies and private for-profit facilities in Myanmar. Oral artemisinin monotherapy (AMT) was available in 27.7% of private sector outlets. Less than 20% across the private sector could correctly state the national first-line treatment for uncomplicated falciparum and vivax malaria [28]. The private sector outlets distributed the oral AMT. The median number of oral AMT tablets was two tablets (about one tenth of a full adult dosage) [29]. *P*. *falciparum* malaria was reduced by 96.5% across the KR2, from 3 827 cases in 2008 to 134 cases in 2016. Besides, the result of our resistance surveillance illustrates that the ACT was still efficacious for the treatment of falciparum malaria [30]. Nowadays, there is not a single promising method to eliminate artemisinin resistant *P*. *falciparum* malaria foci. As a result, a multipronged approach, including the MDA and other expanded treatment (e.g. administration of all confirmed, clinical and suspected cases), is adopted to contain artemisinin resistance [31]. A high coverage of quality-assured treatment could be one of the strategies that prevent the development and spread of artemisinin’s resistance.

In the GFATM supported projects, all positive blood slides and 10% of negative slides were sampled and sent to expert microscopists to reread for lab quality assurance. Results from the experts confirmed that there was an increase in *P*. *vivax* in the KR2, rather than the misjudgement-detection experiences as in Cambodia [32].

The increase in *P*. *vivax* in the KR2 reveals that the control and elimination of *P*. *vivax* malaria is much more difficult than *P*. *falciparum*. Vector control may be less effective in cutting off the transmission of *P*. *vivax* parasites than *P*. *falciparum* because mosquitoes bite early in the evening, obtain blood meals outdoors and rest outdoors in various areas [33]. Although the KR2 had a high coverage and usage rate of insecticide-treated nets (ITNs), especially among the children and pregnant women [13], the incidence of *P*. *viax* malaria still increased from 286 cases in 2012 to 3168 in 2016 after the targets varying from program I to II and III (S1 Table). Other challenges on *P*. *viax* malaria control are: 1) blood-stage *P*. *vivax* often occurs with low parasite densities with gametocytes thus may be misdiagnosed by routine microscopy or RDT as negative; and 2) dormant hypnozoite stage can cause multiple relapses. Furthermore, current diagnostic methods cannot detect the hypnozoites in liver cells and gametocytes of *P*. *vivax* are frequently produced and transmitted to the mosquito before symptoms appearing [33]. Primaquine is the sole available option for killing gametocytes and treating the liver stage parasite but it can cause acute haemolytic anaemia in patients accompanying with severe forms of glucose-6-phosphate dehydrogenase (G6PD) deficiency and cannot be administered to the children under six months of age or pregnant women [33, 34]. Quality assured treatment of all confirmed, clinical and suspected cases can kill dormant hypnozoites and submicroscopic parasites with gametocytes. It may be the main reason that program I (2008–2011) reduced reported *P*. *vivax* malaria from 1 889 cases in 2008 to 400 in 2011.

Two slide positivity peaks occurred in March and June in 2006 while there is only one peak from 2009 to 2015 (from May to July). The change shows that the peak is shrinking and moving. Increasing slide positivity indicates that resurging parasite prevalence is from 2012 to 2016 (S2 Table, S3 Fig). The baseline survey of the sixth GFATM project detected that 17.1% (95%CI: 15.0%-19.2%) of parasite prevalence occurred among community residents [12] and the systemic intervention of the GFATM projects began in early 2008 [5]. In 2006, the first slide positivity peak in March may be the outcome of originally high parasite prevalence. In the area, the population of anopheline mosquitoes starts to boom with rising temperature since April every year. However, breeding sites for *An*. *minimus* is flushed by torrential rain after July decreasing the density of primary vector and reducing the possibility of transmission. Thus, true intensive transmission happens from May to July [35]. After the success of program I (2008–2011) in decreasing the originally high parasite prevalence, the peak of slide positivity turned to high transmission season (i.e. from May to July) in 2009, 2013 and 2015 (see S3 Fig).

Unavoidably, the accuracy of this retrospective research is weakened by a range of factors together with denied access to more abundant data resources. First, the study cannot exclude the impacts of other interventions on malaria burden in the KR2. The ratio of bed net to person reached 1:1.96 (i.e. more than one net for every two people) while the ratio of long-lasting insecticidal net (LLIN) to person was 1:2.52 and 76.1% of people sleeping in LLINs the prior night in 2013 [13]. However, the bed net is not effective in preventing the transmission of *P*. *vivax* due to the barriers mentioned above in the control of *P*. *vivax* malaria [33]. Second, the increasingly unstable political context may also have affected malaria transmission and intervention activities. Since May 12^th^ of 2012, there have been fierce conflicts and struggles between Kachin Independent Army and National Defense Army. It is an ethnical conflict between Kachin ethnics and the mainstream Burmese people. Due to the conflicts, Kachin people left their land and moved to the internally displaced person (IDP) camps next to China-Myanmar borderline. The affected people have nowhere to go but stay in the place due to the inland ethnical conflicts of Myanmar and also the strict border control of China. People moved from a place that had higher levels of malaria transmission could bring parasites with them into the IDP camps. The tenth GFATM project and the department of health of the KIO have set up health facilities while establishing the IDP campus. Although a literature reported that malaria incidence in the IDP camps was significantly lower than the surrounding villages [6], it still seems impossible to totally stop the influx of the political unrest on malaria burden. Third, results of microscopy and RDT were not arbitrated by PCR but only by expert microscopists. But the impacts are even throughout time-series analysis so that the results are still comparable. Fourth, only *P*. *falciparum* and *P*. *vivax* can be defined and discussed in this paper from routine malaria diagnosis, treatment and surveillance because the microscopists who work in routine health facilities have no capacity to accurately identify *P*. *malariae* and *P*. *ovale*. Moreover, the adopted RDT is only able to detect *P*. *falciparum* and *P*. *vivax*. Plus, some *P*. *malariae* and *P*. *ovale* identified by microscopists in lab quality control were included the analysis of *P*. *vivax*. Fifth, this study has not considered whether the patients take medication as required but the compliance of patient is essential to long-treatment course for primaquine. In the GFATM supported projects, directly observed treatment (DOT) was not carried out but health staffs let the caregivers or family members monitoring patients to take drugs in time. An eight day regimen of primaquine (22.5mg/day for adult) was used from 2008 to 2013 and a 14 day regimen of primaquine (15mg/day for adult) was used from 2014 to 2016. Usually, longer medications mean poorer compliance on drugs [36, 37]. Thus, further studies should consider this issue when interpreting the results.

### Conclusions

The results of study demonstrate the importance of high-coverage treatment in the control and elimination of malaria. Treatment scenario adopted in the program I (2008–2011) might be the best in all of the three. A confirmed case-targeted treatment program may miss the infections of submicroscopic parasites, especially for *P*. *vivax*, and then finally fails to treat asymptomatic parasite carriers. Thus, quality-assured treatment of all confirmed, clinical and suspected malaria cases may be helpful for reducing malaria burden and drug resistance of antimalarial drugs.

## Supporting information

S1 Fig Location of study site. (TIF)

S2 Fig Number of persons treated and annual parasite incidence from 2008 to 2016. **API.** Annual parasite incidence. **AFI**. Annual *P*. *falciparum* incidence. **AVI**. Annual *P*. *vivax* incidence. **I.** Start of all confirmed, clinical and suspected cases treated. **II.** Start of confirmed and clinical cases treated. **III**. Start of only confirmed cases treated.
(TIF)

S3 Fig Seasonal fluctuation of slide positivity in 2006, 2009, 2013 and 2015. (TIF)

S1 Table Changes of malaria burden and treatment targeted in Kachin Special Region II, Myanmar from 2008 to 2016. (DOC)

S2 Table Changes of slide positivity of febrile patients at Laiza Hospital, Kachin Special Region II, Myanmar from 2008 to 2016. (DOC)
