# Supplementary figures and images for: Malaria burden and treatment targets in Kachin Special Region II, Myanmar from 2008 to 2016: A retrospective analysis
Source: PLoS One. 2018 Apr 3;13(4):e0195032. doi: 10.1371/journal.pone.0195032 (PMC5882093; doi:10.1371/journal.pone.0195032)

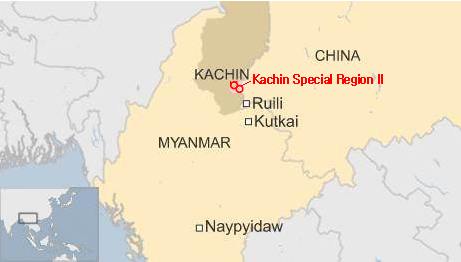

Supplement: S1 Fig — (TIF) [file pone.0195032.s001.TIF]

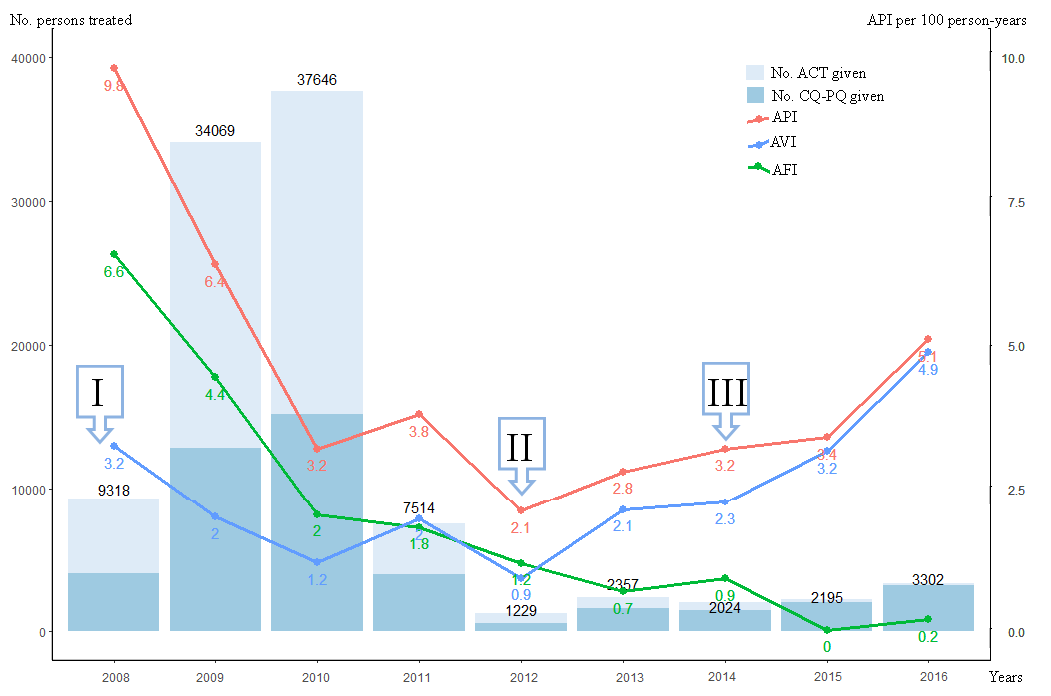

Supplement: S2 Fig — API. Annual parasite incidence. AFI. Annual P. falciparum incidence. AVI. Annual P. vivax incidence. I. Start of all confirmed, clinical and suspected cases treated. II. Start of confirmed and clinical cases treated. III. Start of only confirmed cases treated. (TIF) [file pone.0195032.s002.tif]

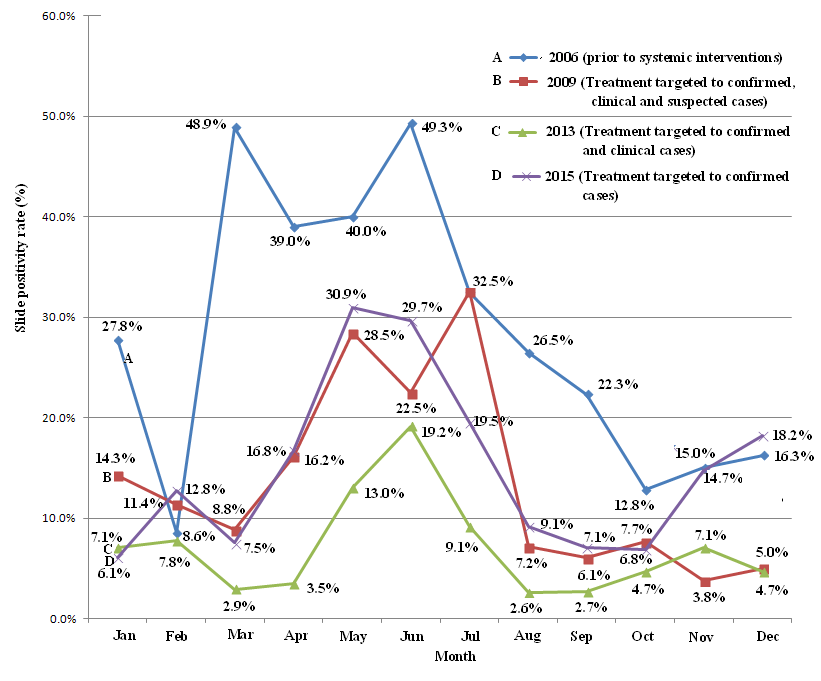

Supplement: S3 Fig — (TIF) [file pone.0195032.s003.TIF]
