# Supplementary material for: Malaria burden and treatment targets in Kachin Special Region II, Myanmar from 2008 to 2016: A retrospective analysis
Source: PLoS One. 2018 Apr 3;13(4):e0195032. doi: 10.1371/journal.pone.0195032 (PMC5882093; doi:10.1371/journal.pone.0195032)
Supplement: S1 Table — (DOC) [file pone.0195032.s004.doc]

Table 1 Changes of malaria burden and treatment targeted in Kachin Special Region II, Myanmar from 2008 to 2016

| **Year** | **Person-years** | ***Pf* (%, 95%CI)** | ***Pv* (%, 95%CI)** | **Total cases** | **API (95%CI) per 100 person-years** | **IRR( 95%CI)** | **P value** | **No. ACT given** | **No. CQ-PQ given** | **No. persons treated (% of the entire population, 95%CI)** |
| --- | --- | --- | --- | --- | --- | --- | --- | --- | --- | --- |
| **China R6/Program I (2008-2011): All confirmed, clinical and suspected cases treated.** | | | | | | | | | | |
| 2008 | 58229 | 3827(67.0, 65.7-68.2） | 1889(33.0, 31.8-34.3） | 5716 | 9.8( 9.6-10.1) | 1 |  | 5310 | 4008 | 9318 (16.0, 15.7-16.3) |
| 2009 | 59176 | 2623(68.8, 67.3-70.3） | 1178(30.9, 29.4-32.4） | 3811 | 6.4(6.2-6.6) | 0.66 (0.63-0.66) | <0.0001 | 21255 | 12814 | 34069 (57.6, 57.2-58.0) |
| 2010 | 60123 | 1223(62.8, 60.7-65.0） | 716(36.8, 34.6-39.0） | 1946 | 3.2(3.1-3.4) | 0.33 (0.31-0.35) | <0.0001 | 22457 | 15189 | 37646 (62.6, 62.2-63.0) |
| 2011 | 20362 | 368(47.1, 43.5-50.7） | 400(51.2, 47.5-54.7） | 782 | 3.8(3.6-4.1) | 0.39 (0.36-0.42) | <0.0001 | 3530 | 3984 | 7514 (12.3, 12.0-12.6) |
| **China R10/Program II (2012-2013): Confirmed and clinical cases treated** | | | | | | | | | | |
| 2012 | 31031 | 368(56.3, 52.4-60.1） | 286(43.7, 39.9-47.6） | 654 | 2.1(2.0-2.3) | 1 |  | 692 | 537 | 1229 (2.0, 1.9-2.1) |
| 2013 | 63117 | 435(24.5, 22.5-26.6） | 1339(75.5, 73.4-77.5） | 1774 | 2.8(2.7-2.9) | 1.33 (1.22-1.46) | <0.0001 | 766 | 1591 | 2357 (3.7, 3.6-3.9) |
| **Myanmar GFNFM/Program III (2014-2016): Only confirmed cases treated.** | | | | | | | | | | |
| 2014 | 63748 | 586(29.0, 27.0-31.0） | 1438(70.0, 69.0-73.0） | 2024 | 3.2(3.0-3.3) | 1 |  | 586 | 1438 | 2024 (3.2, 3.0-3.3) |
| 2015 | 64386 | 165(7.5, 6.4-8.7） | 2030(92.5, 91.3-93.6） | 2195 | 3.4(3.3-3.6) | 1.01 (1.07-1.14) | 0.0197 | 165 | 2030 | 2195 (3.4, 3.3-3.6) |
| 2016 | 65030 | 134(4.1, 3.4-4.8） | 3168(95.9, 95.2-96.5） | 3302 | 5.1( 4.9-5.2) | 1.60 (1.51-1.69) | <0.0001 | 134 | 3168 | 3302 (5.1, 4.9-5.2) |

Notes: *1) Pf = Plasmodium falciparum, Pv= P. vivax,* Total cases include *Pf，Pv，P. malariae* and *P ovale*; 2) CI=Confidence interval，API= annual parasite incidence，IRR= API ratio; 3) ACT= artemisinin-based combination therapy; CQ-PQ =chloroquine-primaquine; 4) The GFATM stopped its Chinese operations between May and December, 2011, the Information System in KR2 Myanmar established and maintained by the GFATM grants, could run only for four months (January to April), n= 4 X 61086/12 =20362 person-years. In 2012, The GFATM project spent the first half of the year to recruit new staff and restore project activities, n=6 x 62062/12 =31031 person-years. 5) GFNFM = Global Fund New Funding Model.
