# Supplementary material for: Malaria burden and treatment targets in Kachin Special Region II, Myanmar from 2008 to 2016: A retrospective analysis
Source: PLoS One. 2018 Apr 3;13(4):e0195032. doi: 10.1371/journal.pone.0195032 (PMC5882093; doi:10.1371/journal.pone.0195032)
Supplement: S2 Table — (DOC) [file pone.0195032.s005.doc]

Table 2 Changes of slide positivity of febrile patients at Laiza Hospital, Kachin Special Region II, Myanmar from 2008 to 2016

| **Year** | **No. tested** | ***Pf* (%, 95%CI)** | ***Pv* (%, 95%CI)** | **Total positivity** | **SPR% (95%CI)** | **RR( 95%CI)** | **P value** |
| --- | --- | --- | --- | --- | --- | --- | --- |
| **China R6/ Program I (2008-2011): All confirmed, clinical and suspected cases treated.** | | | | | | | |
| 2008 | 1135 | 64 (25.9, 20.6-31.8） | 183 (74.1, 68.2-79.4） | 247 | 21.8 ( 19.4-24.3) | 1 |  |
| 2009 | 1400 | 42 (23.2, 17.3-30.0） | 139 (76.8, 70.0-82.7） | 181 | 12.9 (11.2-14.8) | 0.59 (0.50-0.71) | <0.0001 |
| 2010 | 2206 | 136 (75.1, 68.2-81.3） | 42 (23.2, 17.3-30.0） | 181 | 8.2 (7.1-9.4) | 0.38 (0.32-0.45) | <0.0001 |
| 2011 | 2459 | 50 (40.7, 31.9-49.9） | 73 (59.3, 50.1-68.1） | 123 | 5.0 (4.2-5.9) | 0.23 (0.19-0.28) | <0.0001 |
| **China R10/ Program II (2012-2013): Confirmed and clinical cases treated.** | | | | | | | |
| 2012 | 2246 | 40 (31.3, 23.4-40.0） | 87 (68.0, 59.1-75.9） | 128 | 5.7 (4.8-6.7) | 1 |  |
| 2013 | 3720 | 61 (22.6, 17.7-28.1） | 209 (77.4, 71.9-82.3） | 270 | 7.3 (6.4-8.2) | 1.27 (1.04-1.56) | 0.0194 |
| **Myanmar GFNFM/Program III (2014-2016) : Only confirmed cases treated** | | | | | | | |
| 2014 | 3933 | 31 (9.4, 6.5-13.1） | 298 (90.6, 86.9-93.5） | 329 | 8.4 (7.5-9.3) | 1 |  |
| 2015 | 3903 | 4 (0.7, 0.2-1.7） | 607 (99.2, 98.1-99.7） | 612 | 21.1 (19.6-22.6) | 1.87 (1.65-2.13) | <0.0001 |
| 2016 | 4938 | 19 (1.5, 0.9-2.4） | 1230 (98.5, 97.6-99.1） | 1249 | 25.3 (24.1-26.5) | 3.02 (2.70-3.39) | <0.0001 |

Notes: 1) *Pf = Plasmodium falciparum, Pv= P. vivax,* Total positivity includes *Pf，Pv，P. malariae*, *P ovale* and mixed; 2) CI=Confidence interval，SPR= Slide positivity rate，RR= risk ratio. 3) GFNFM = Global Fund New Funding Model
